# Effect of gonial angle variation on stress distribution and fracture susceptibility in the mandibular angle: a finite element analysis

**DOI:** 10.1186/s12903-026-09234-1

**Published:** 2026-07-27

**Authors:** Tevfik KIZILSEKİ, Halil İbrahim DURMUŞ

**Affiliations:** Department of Oral and Maxillofacial Surgery, Faculty of Dentistry, Adıyaman University, Adıyaman, Türkiye

**Keywords:** Angle fracture, Mandibular fracture, Gonial angle, Finite element analysis

## Abstract

**Aim:**

The mandibular angle is affected by morphological and functional factors. This study aimed to evaluate the stress distribution and susceptibility to fracture in the angle region under two different traumatic loading conditions in mandibular models with different gonial angles using finite element analysis.

**Materials and methods:**

A mandible model with three different gonial angles (115°, 125°, and 135°) was created based on a standard reference representing human anatomy. In the linear static analysis, a traumatic force of 2000 N was applied to a 1 cm diameter circular contact area in two different loading scenarios: (i) frontally, perpendicular to the coronal plane, and to the symphysis region, and (ii) laterally to the angulus region. The finite element analysis was performed on a workstation with an Intel^®^ Xeon™ 2.4 GHz processor and 48 GB of RAM. Mimics was used for segmentation, HyperMesh for mesh generation, Abaqus/Standard for analysis, and Abaqus/CAE for visualization of the results.

**Results:**

In both loading scenarios, as the gonial angle increased (115° → 125° → 135°), the stress concentration in the mandibular angulus region decreased. The highest stress values were found in the 115° model, whereas the lowest values were found in the 135° model. When the principal stress and Von Mises analyses were evaluated together, a more pronounced stress concentration was observed in the angulus region in models with a narrower gonial angle.

**Conclusion:**

Within the limitations of this finite element analysis study, lower gonial angle models demonstrated higher stress concentrations in the mandibular angulus region under the simulated loading conditions. These findings suggest a potential biomechanical tendency toward increased stress localization rather than a direct prediction of clinical fracture risk. Further experimental and clinical studies are required to validate these observations and determine their clinical significance.

## Introduction

Mandibular angular fractures are a frequently occurring group of mandibular fractures that are of particular importance in treatment planning because of their stability and fragment displacement. The main factors determining fracture localization in the mandible are the direction and intensity of the traumatic force, bone morphology, and biomechanical loads created by the masticatory muscles [1]. Mandibular fractures are caused by internal biomechanical effects and external forces [2]. In maxillofacial trauma series, mandibular fractures are among the most frequently encountered fractures in clinical practice [3] and show a significant concentration, especially in the young adult age group [4]. Angular fractures have been reported in approximately 30% of cases. This distribution suggests that some areas of the mandible are anatomically and functionally more susceptible to stress concentration [5].

The angulus region is considered a critical area because it is the transition zone between the toothed segment and ramus [6]. An angle fracture is characterized by a typical fracture line that begins distal to the second molar and extends through the retromolar region and the inferior-posterior borders of the ramus [7]. However, explaining these fractures solely through local anatomical features is insufficient; it has been suggested that individual morphological differences can also significantly influence predisposition to fracture initiation. In this context, the gonial angle stands out as a parameter that reflects mandibular morphology and is related to growth direction. The gonial angle is defined by the intersection of the ramus line and the line representing the lower mandibular border [8] and can increase or decrease depending on the rotation pattern of the mandible [9]. It has been reported that in a high gonial angle pattern, the functional efficiency of the chewing muscles may decrease, the biting force may be reduced, and, as a result of the accompanying adaptive changes, the cortical bone thickness in the angulus region may decrease [6, 10]. Three-dimensional anatomical dissections and ultrasonographic studies have yielded findings supporting these muscle-based biomechanical differences [7, 11].

Clinical data indicate that individuals with a high gonial angle have a significantly increased likelihood of developing an angulus fracture, and the risk is considerably higher than that in individuals with a normal or low gonial angle pattern [7]. At first glance, the present findings appear to contrast with clinical studies reporting a higher incidence of mandibular angle fractures in individuals with wider gonial angles. However, stress concentration and fracture incidence do not necessarily represent the same biomechanical phenomenon. The finite element model used in this study evaluates the local mechanical response of bone under standardized loading conditions, whereas clinical fracture occurrence is influenced by multiple additional factors including cortical bone thickness, bone density, muscle function, impact direction, and trauma energy. Therefore, higher stress concentration in low gonial angle models may reflect a greater tendency for localized stress accumulation, while the increased fracture incidence reported in high gonial angle individuals may be associated with reduced structural resistance of the mandibular angle complex. However, the relationship between the gonial angle and angulus fracture has largely been defined through clinical and radiological observations; studies aimed at quantitatively explaining this relationship based on three-dimensional stress distribution are limited [6, 7]. Because measuring stress distribution in the mandible in vivo is limited from a practical and ethical standpoint [12], finite element analysis offers a significant advantage in investigating the effect of morphological variations, such as the gonial angle, on the mechanical behavior of the angulus region.

This study aimed to investigate the effects of different gonial angle configurations on stress distribution in the mandibular angle region using finite element analysis. Through analyses performed on three-dimensional mandibular models under standardized loading conditions, we aimed to quantitatively determine the biomechanical role of the gonial angle in susceptibility to angular fractures.

## Materials and methods

### Finite element analysis workflow and software

Finite element analyses were performed on a workstation with an Intel^®^ Xeon™ 2.4 GHz processor and 48 GB of RAM. Mimics (Materialise NV, Leuven, Belgium) was used for segmentation, and HyperMesh (Altair Engineering Inc., Troy, MI, USA) was used for mesh generation and preprocessing. The analysis was performed using Abaqus/Standard (Dassault Systèmes, Vélizy-Villacoublay, France), and the results were visualized using Abaqus/CAE. 3-matic software was used to edit and preprocess the STL surface data.

### Creating three-dimensional models

**Fig. 1:**
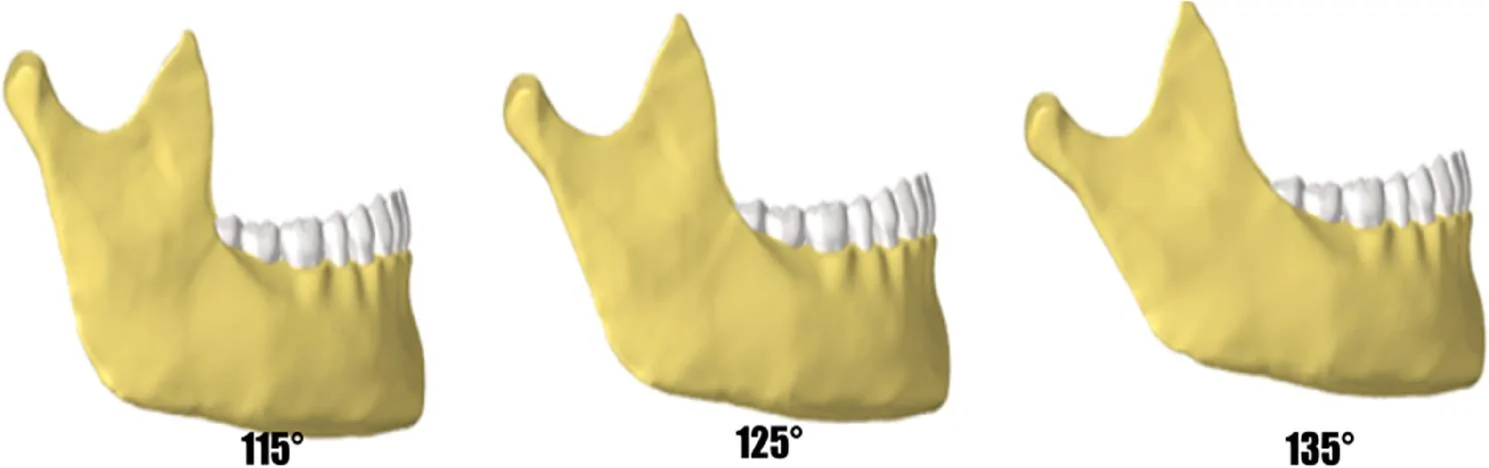
Mandible models with different gonial angles (115°, 125°, and 135°)

The initial mandibular geometry was generated using Mimics software (Materialise NV, Leuven, Belgium) based on a standard digital anatomical reference representing average human mandibular anatomy [13, 14]. The resulting STL model was subsequently imported into 3-matic (Materialise NV, Leuven, Belgium), where surface optimization and geometric preprocessing procedures were performed before transferring the models to HyperMesh for finite element mesh generation. Gonial angle variations (115°, 125°, and 135°) were created by changing only the geometric configuration of the angulus region on the same reference mandibular geometry. The resulting mandibular models with different gonial angles are shown in Fig. 1. During this process, the symphysis, alveolar crest, condylar head, and dental arch form were preserved; this approach was intentionally adopted to isolate the biomechanical effect of gonial angle variation. However, it should be acknowledged that in vivo mandibular morphologies associated with different gonial angle patterns may also exhibit variations in ramus height, cortical thickness, mandibular body dimensions, and bone quality. Therefore, the present models represent a controlled geometric comparison rather than the full spectrum of anatomical variation observed clinically.

### Material properties

All structures were included in the analyses, assuming homogeneous, isotropic, and linear elastic properties. Furthermore, all interfaces between cortical bone, cancellous bone, teeth, and periodontal ligament were defined as bonded contacts. Therefore, no relative displacement, separation, or sliding was permitted between adjacent structures during loading. The mechanical properties assigned to each tissue are presented in Table 1. Accordingly, the following values were defined: E = 13,700 MPa, ν = 0.30 for cortical bone; E = 1,370 MPa, ν = 0.30 for spongy bone; E = 19,600 MPa, ν = 0.31 for tooth tissue (enamel + dentin); and E = 50 MPa, ν = 0.45 for the periodontal ligament.

**Table 1:** The mechanical properties of the tissues were defined in the model

Tissue / Material	Modulus of Elasticity (E) [MPa]	Poisson’s Ratio (ν)
Cortical Bone	13,700	0.30
Cancellous Bone	1,370	0.30
Tooth (enamel + dentin)	19,600	0.31
Periodontal ligament	50	0.45

The material properties assigned to the cortical bone, cancellous bone, tooth structure, and periodontal ligament were adopted from previously published finite element studies and represent average mechanical characteristics of human mandibular tissues. Sex-specific differences in bone density, cortical thickness, and elastic properties were not incorporated into the present models. Therefore, the assigned material parameters should be interpreted as generalized values rather than male- or female-specific biomechanical properties. Previous finite element investigations evaluating sex-related differences in mandibular bone mechanics have reported that although variations in cortical bone properties may influence displacement behavior, the overall stress distribution patterns remain largely unchanged between male and female models. Therefore, generalized material properties were adopted in the present study to isolate the biomechanical influence of gonial angle variation while minimizing additional sources of variability [15].

### Finite element modeling and mesh generation

**Fig. 2:**
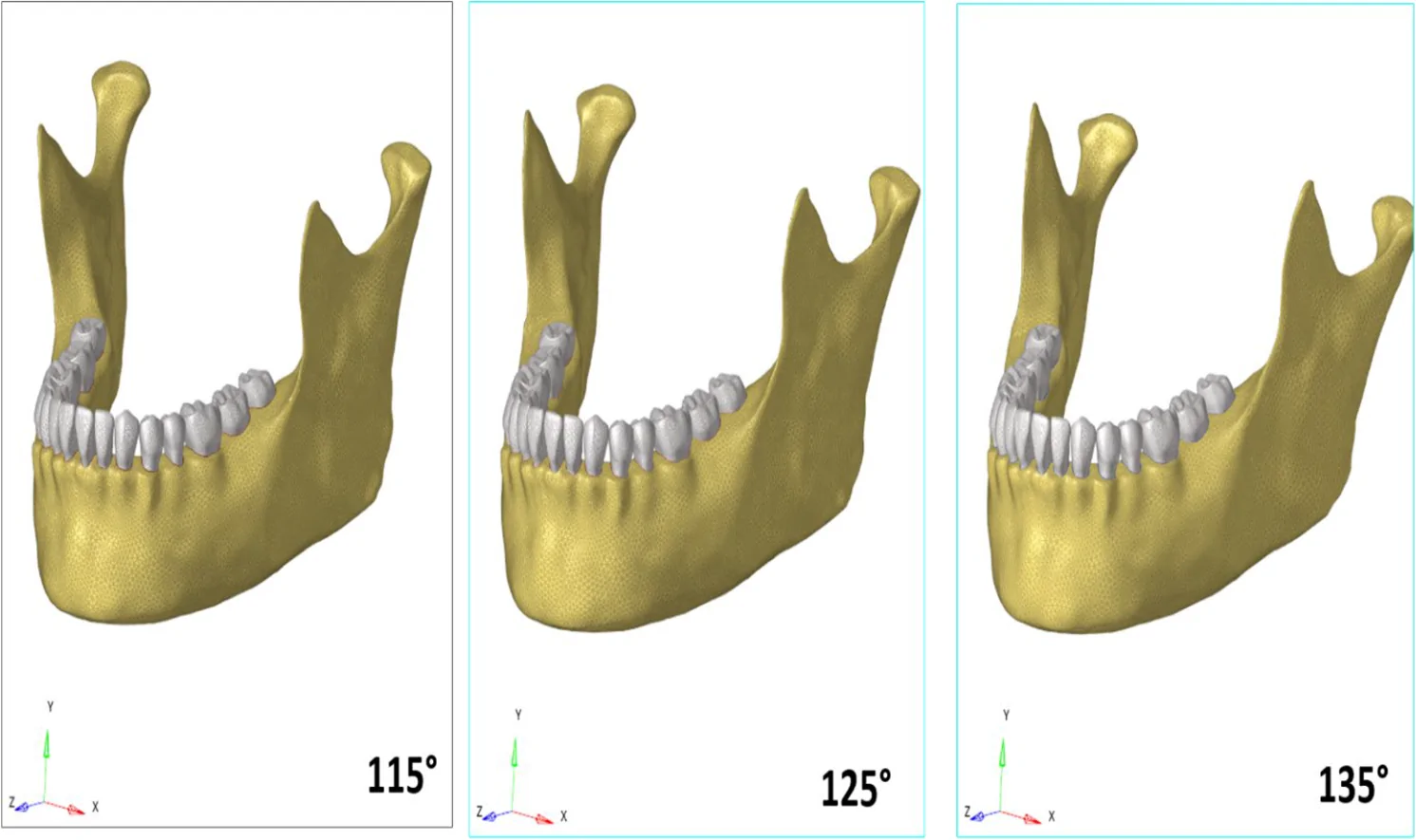
Finite element mesh of the mandibular model generated for biomechanical analysis

The prepared mandibular models were adapted for finite element analysis, imported into Abaqus software, and meshing was performed using tetrahedral elements. Smaller element sizes are preferred to obtain more accurate results for the cortical bone and tooth structures [16–18]. Preliminary mesh refinement assessments were performed during model development. Further reduction of element size did not result in substantial changes in the overall stress distribution patterns; therefore, the selected mesh density was considered sufficient for comparative biomechanical analysis. The numbers of elements and nodes for each model are summarized in Table 2. The final mesh structure comprised three models: the model with a 115° gonial angle, which had 757,622 elements and 138,131 nodes, the model with a 125° gonial angle, which had 759,473 elements and 138,763 nodes; and the model with a 135° gonial angle, which had 916,200 elements and 164,926 nodes. The element type was defined as tetrahedral in all models. The finite element mesh generated for biomechanical analysis is shown in Fig. 2. Following mesh generation, the finite element meshes were inspected for geometric consistency and element distortion. No severely distorted elements were identified, and the final mesh quality was considered suitable for numerical analysis.

**Table 2:** Number of elements and nodes in models

Model	Number of Element	Number of Nodes	Element Type
115°model	757,622	138,131	Tetrahedral
125° model	759,473	138,763	Tetrahedral
135° model	916,200	164,926	Tetrahedral

(The element type was used as tetrahedral in all models.)

### Border conditions and loading scenarios

By fixing the condylar regions at all degrees of freedom (6 DOF), a rigid support boundary condition was defined, and the same boundary conditions were standardized across all models to ensure the reliability of inter-model comparisons. In each model, two different loading scenarios of 2000 N were applied through a circular contact area with a diameter of 1 cm in the symphyseal region. The loading magnitude of 2000 N was selected based on previous finite element studies investigating mandibular trauma biomechanics and high-energy impact conditions [19–22]. This load level has been widely used to simulate traumatic forces under standardized experimental conditions.

**Fig. 3:**
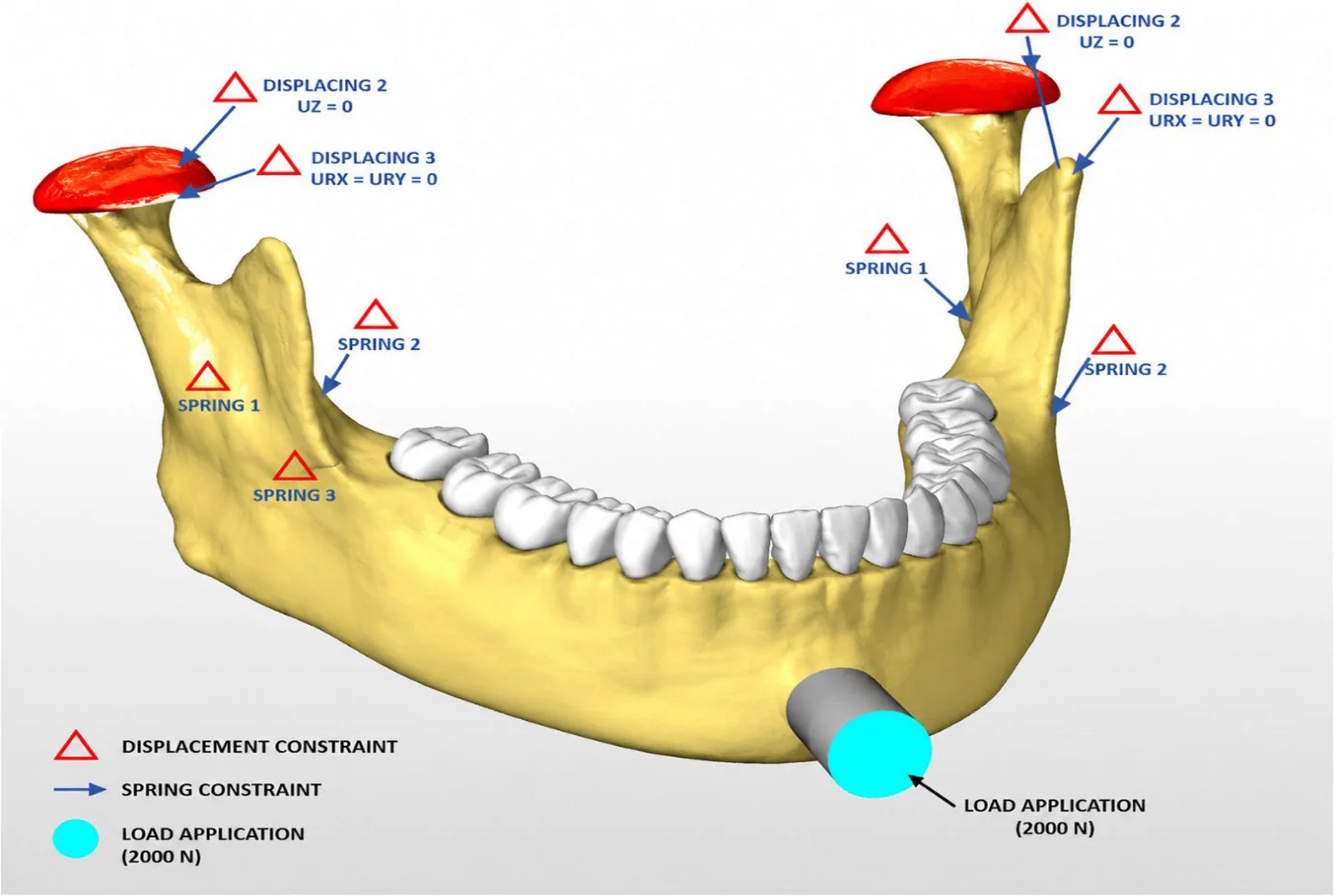
Frontal loading applied to the symphysis region

**Fig. 4:**
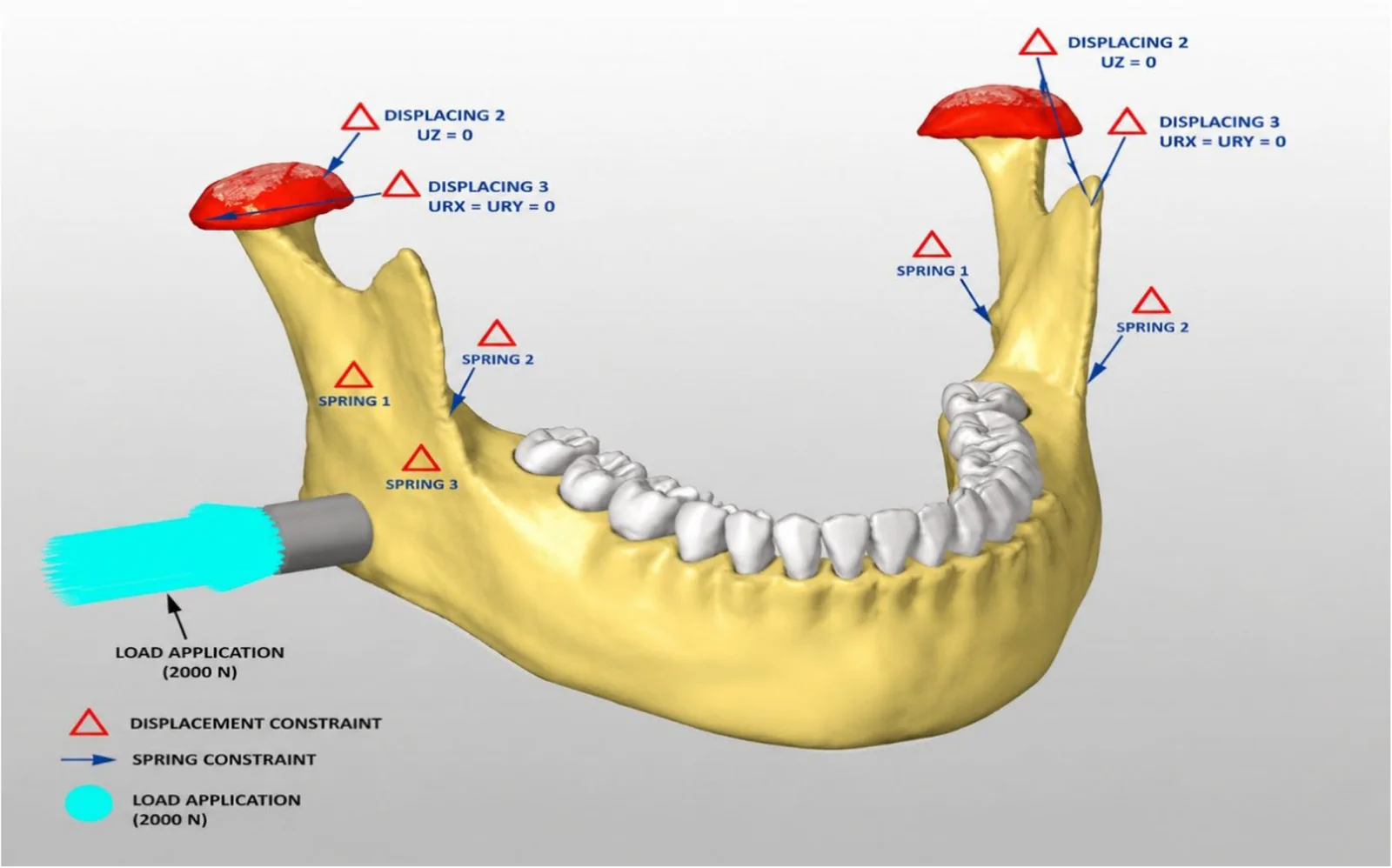
Lateral loading to the angulus region

1. Symphysis region loading: 2000 N in the frontal direction perpendicular to the coronal plane **(**Fig. 3**)**.
2. Angular region loading: 2000 N from outside to inside (lateral direction) **(**Fig. 4**)**.

## Results

### Evaluation criteria

In this study, an absolute fracture threshold was not defined; instead, the obtained stress values were interpreted as relative biomechanical indicators of susceptibility to fracture initiation. Stress distributions were evaluated according to Signed Von Mises, maximum principal stress (Pmax), and minimum principal stress (Pmin) criteria. The use of signed von Mises stress allows differentiation between tensile- and compressive-dominated stress states by incorporating the sign of the dominant principal stress into the equivalent stress measure. Unlike conventional von Mises stress, which provides only magnitude without directional information, the signed approach enables a more comprehensive interpretation of biomechanical behavior, particularly in regions subjected to bending, such as the mandibular angulus. This distinction is important for identifying tensile stress concentrations, which are more relevant to crack initiation and fracture development. The stress distributions after each loading scenario were compared between the models, with particular attention to the mandibular angulus region.

### Symphysis region – frontal loading (2000 N)

**Fig. 5:**
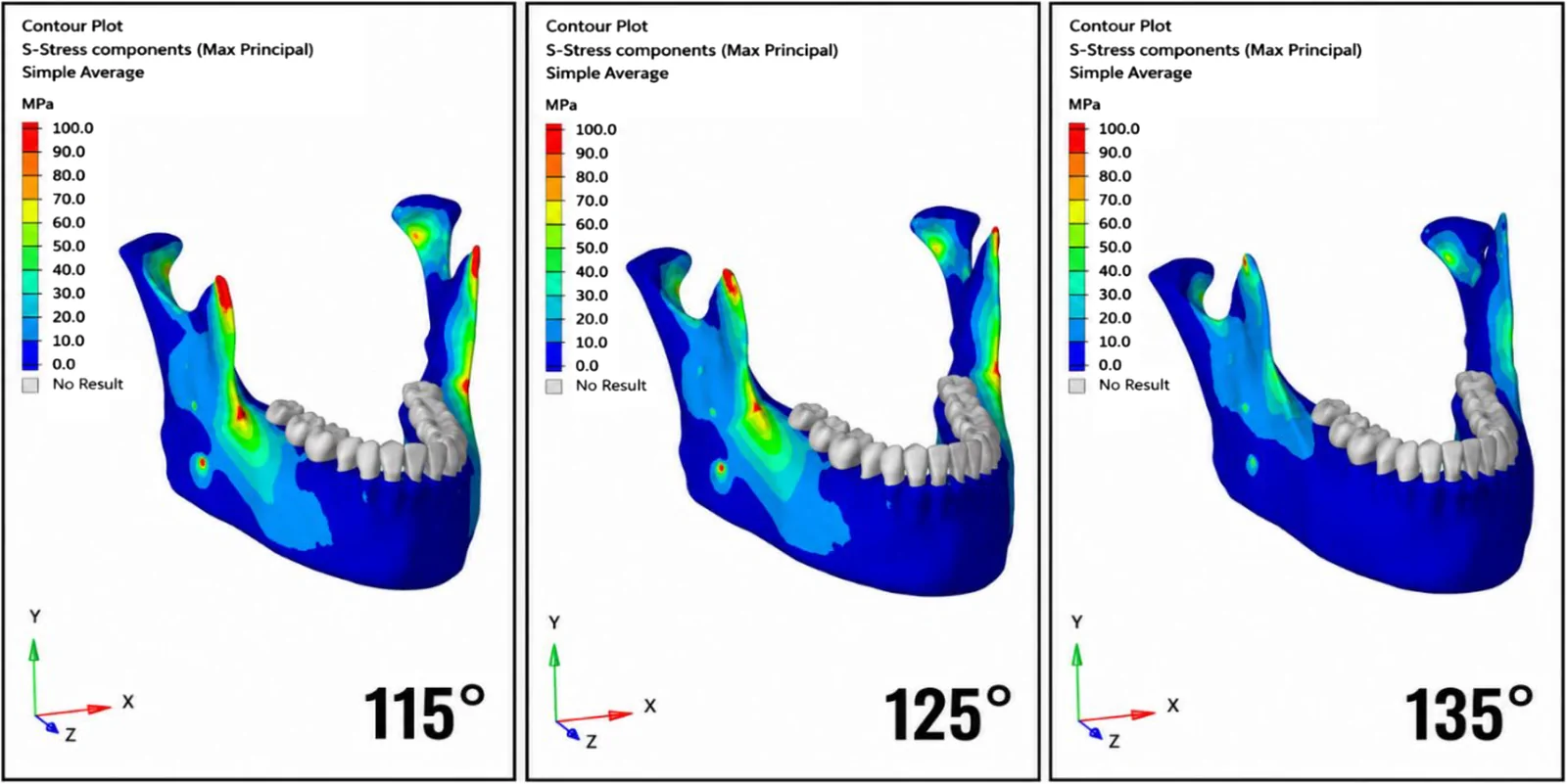
Distribution of the maximum principal stress (Pmax) after frontal loading (2000 N) applied to the symphysis region in mandibular models with different gonial angles (115°, 125° and 135°)

**Fig. 6:**
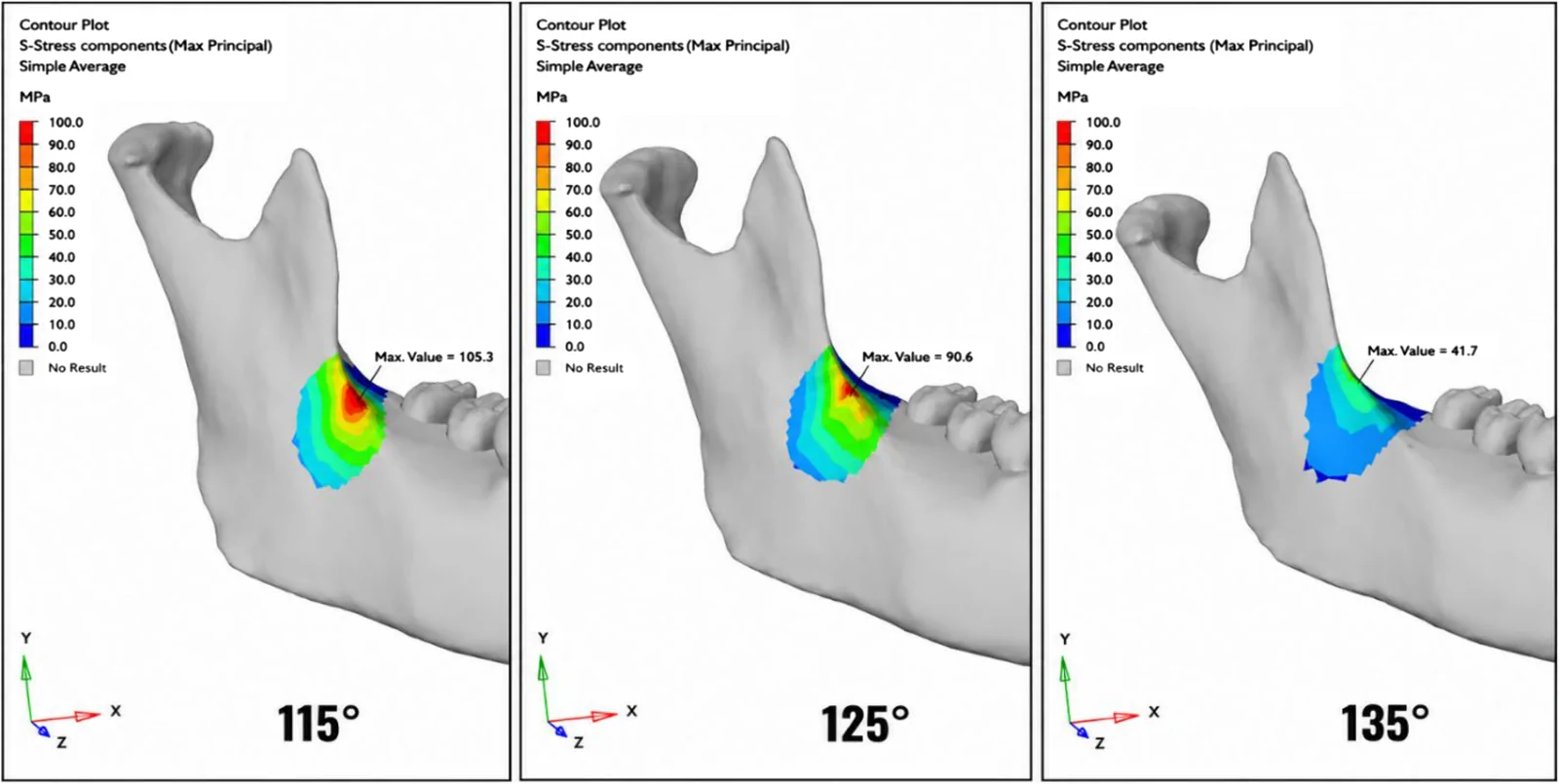
Localized distribution of the maximum principal stress (Pmax) in the mandibular angle region after frontal loading (2000 N) applied to the symphysis region. (115°, 125° and 135°)

**Fig. 7:**
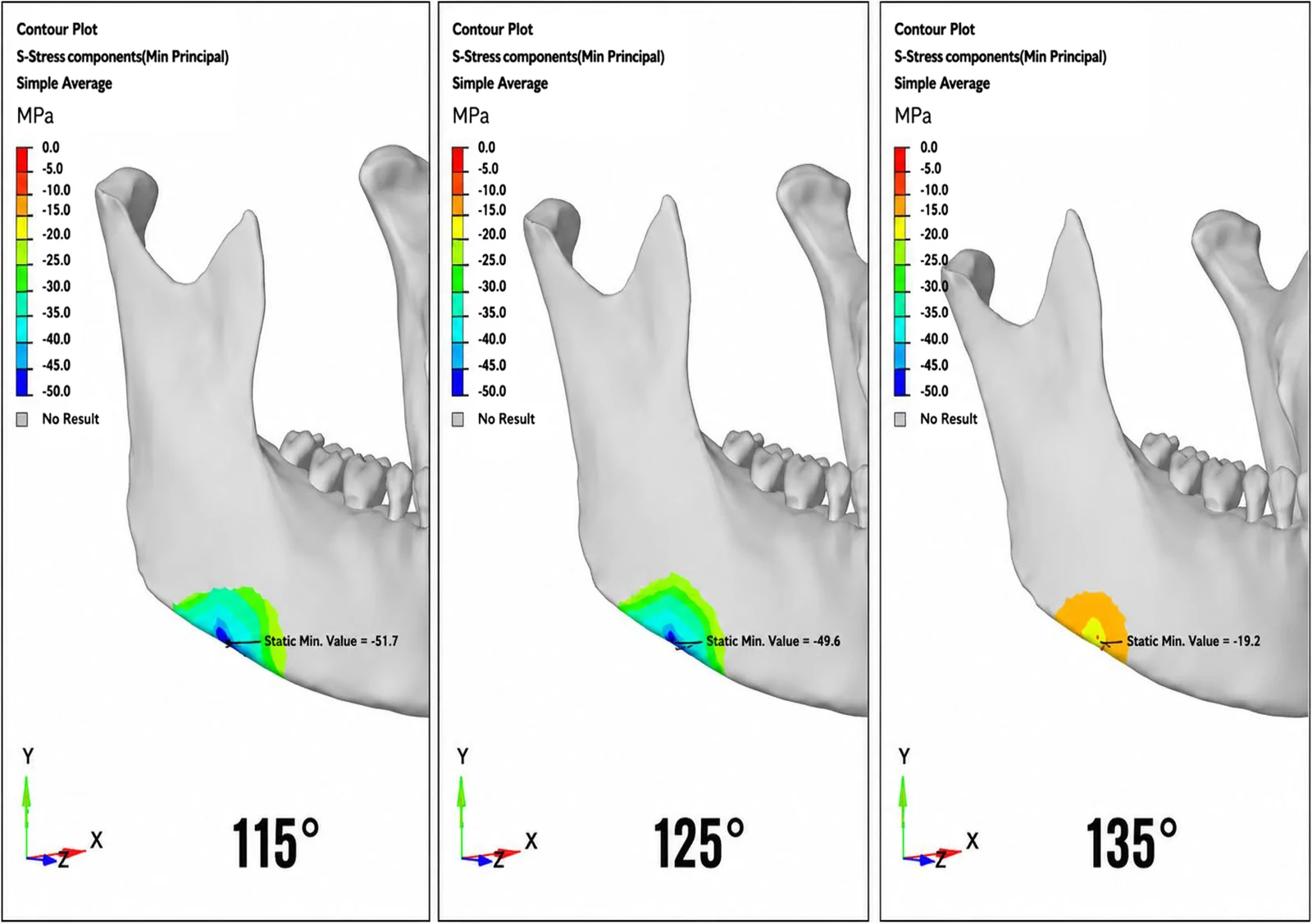
Distribution of the minimum principal stress (Pmin) after frontal loading (2000 N) applied to the symphysis region in mandibular models with different gonial angles (115°, 125° and 135°)

**Fig. 8:**
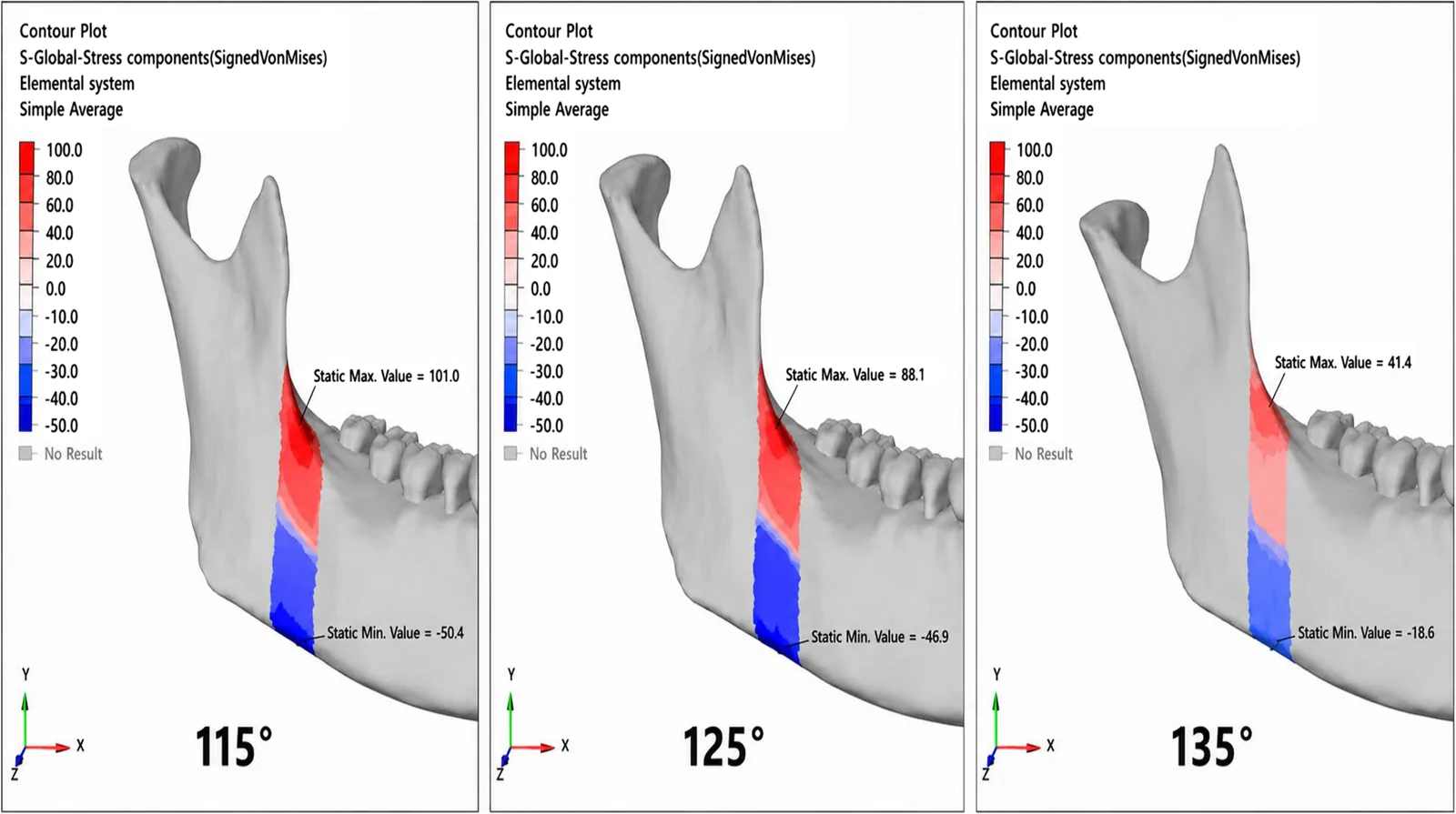
Signed von Mises stress distribution after frontal loading (2000 N) applied to the symphysis region in mandibular models with different gonial angles (115°, 125° and 135°)

Under a 2000 N load applied to the symphysis region in the frontal direction, stress accumulation in the angulus region decreased as the gonial angle increased. The highest stress values were observed in the 115° model, where the Signed Von Mises and tensile stresses (Pmax) were spread over a wider area. In the 125° model, the stress distribution became more balanced, and in the 135° model, the Signed Von Mises, Pmax, and Pmin values decreased significantly, localizing the stress to a more limited area. Furthermore, it was determined that compressive stresses (Pmin) were concentrated in the inferior cortex of the angulus in the 115° model, and that these pressure zones narrowed as the gonial angle increased, as summarized in Table 3 and illustrated in Figs. 5, 6, 7 and 8.

**Table 3:** Pmax, pmin, and signed von mises stresses obtained after frontal loading (2000 N) of the symphysis

Model (Gonial Angle)	Pmax (MPa)	Pmin (MPa)	Signed Von Mises (MPa) (+) MPa (-) MPa	
115° model	105.3	−51.7	101.1	-50.4
125° model	90.6	−49.6	88.1	-46.9
135° model	41.7	−19.2	41.4	-18.6

The signed von Mises values represent the von Mises equivalent stress, indicated by a (+/−) sign depending on the direction of the dominant principal stress. Therefore, both positive (maximum) and negative (minimum) values were reported separately in the analysis results

### Angular region – lateral loading (2000 N)

**Fig. 9:**
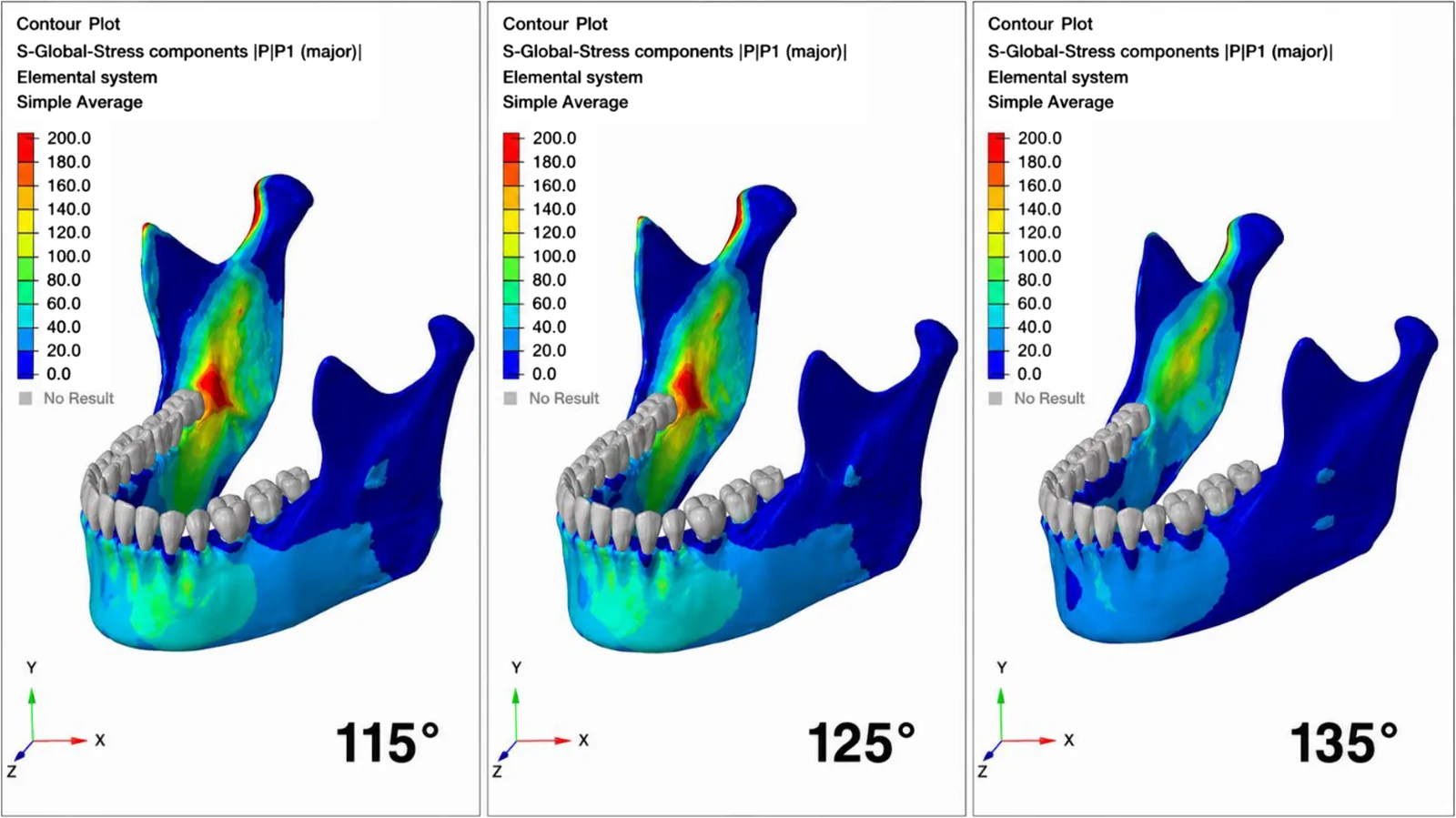
Generalized distribution of the maximum principal stress (Pmax) after lateral loading (2000 N) applied to the angular region in mandibular models with different gonial angles (115°, 125° and 135°)

**Fig. 10:**
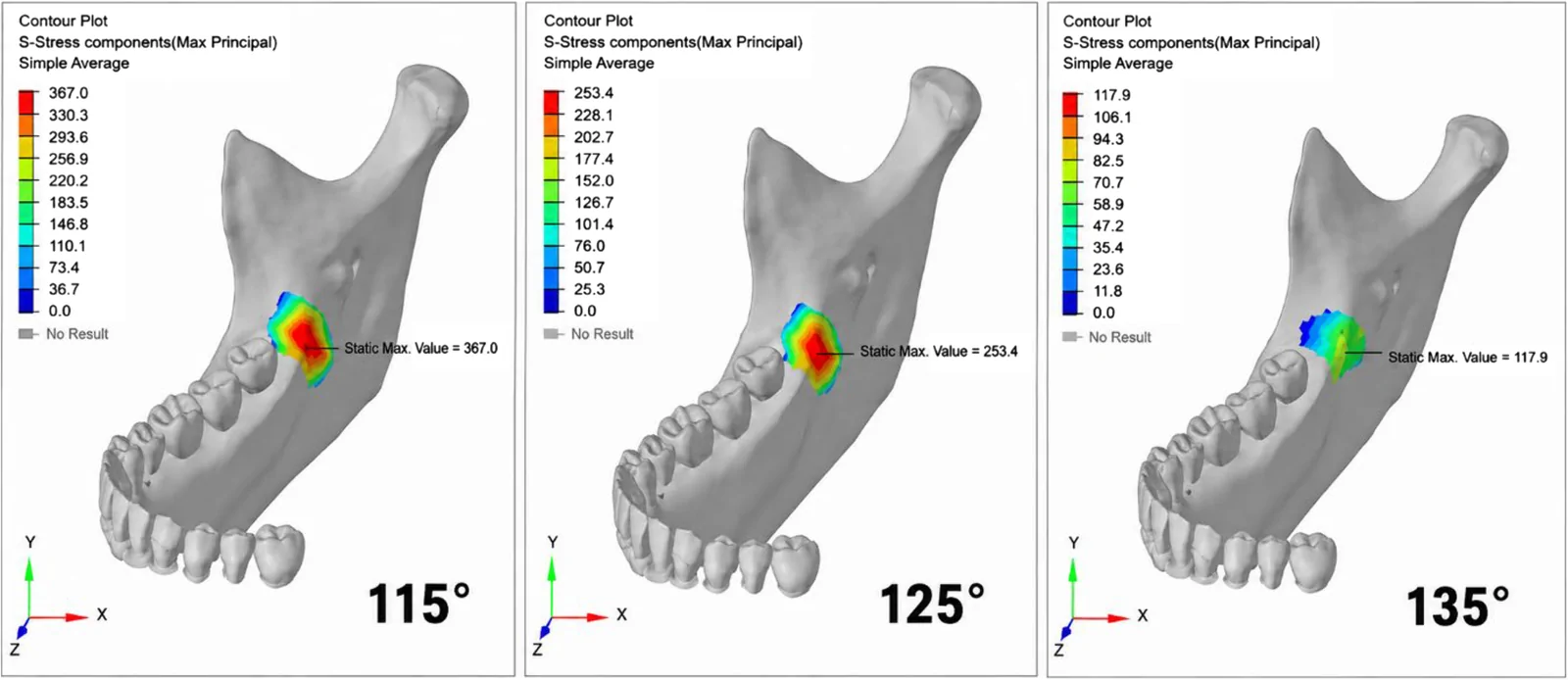
Localized distribution of the maximum principal stress (Pmax) in the mandibular angle region after lateral loading (2000 N) applied to the angular region in mandibular models with different gonial angles (115°, 125° and 135°)

**Fig. 11:**
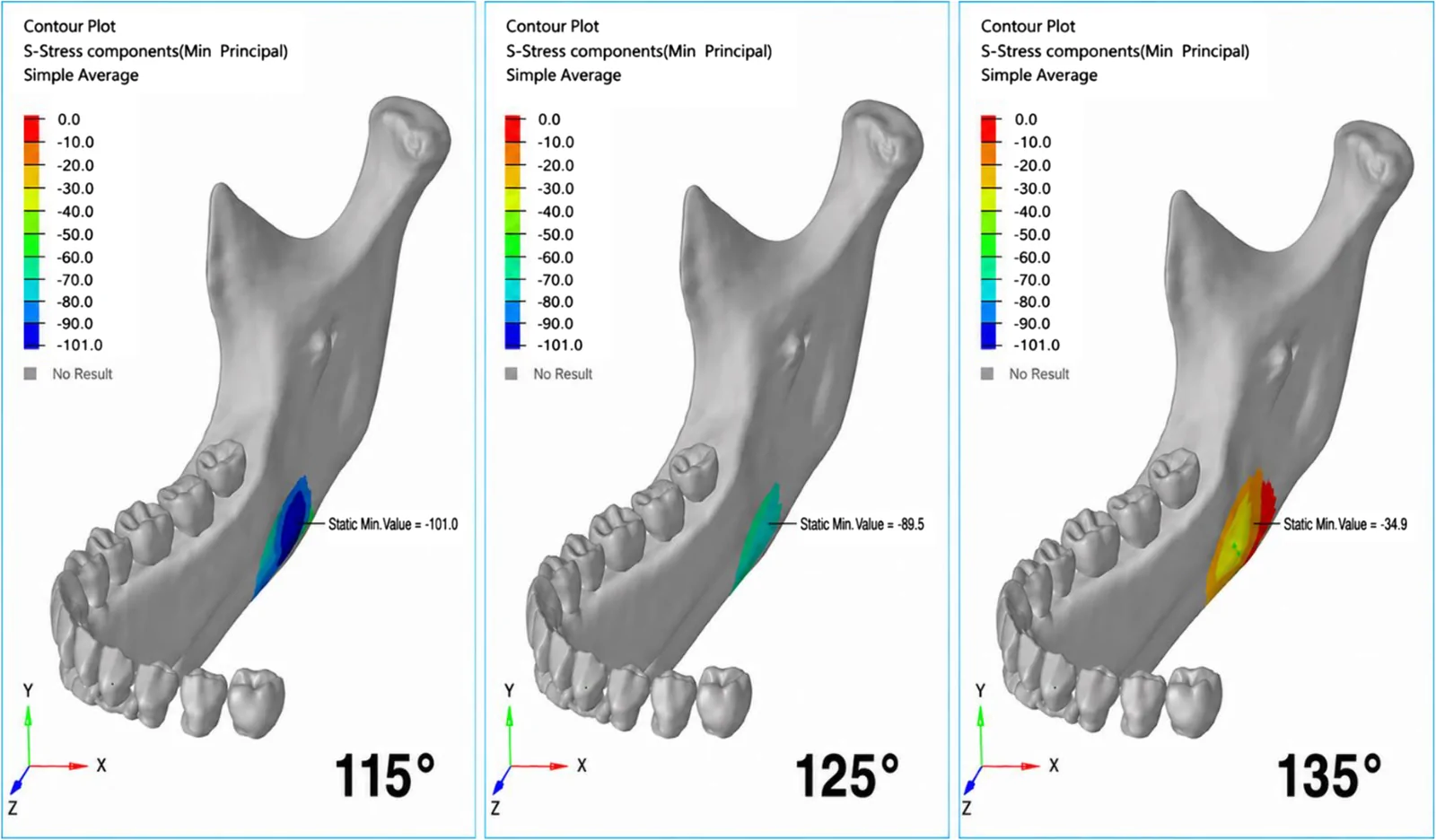
Distribution of the minimum principal stress (Pmin) after lateral loading (2000 N) applied to the angular region in mandibular models with different gonial angles (115°, 125° and 135°)

**Fig. 12:**
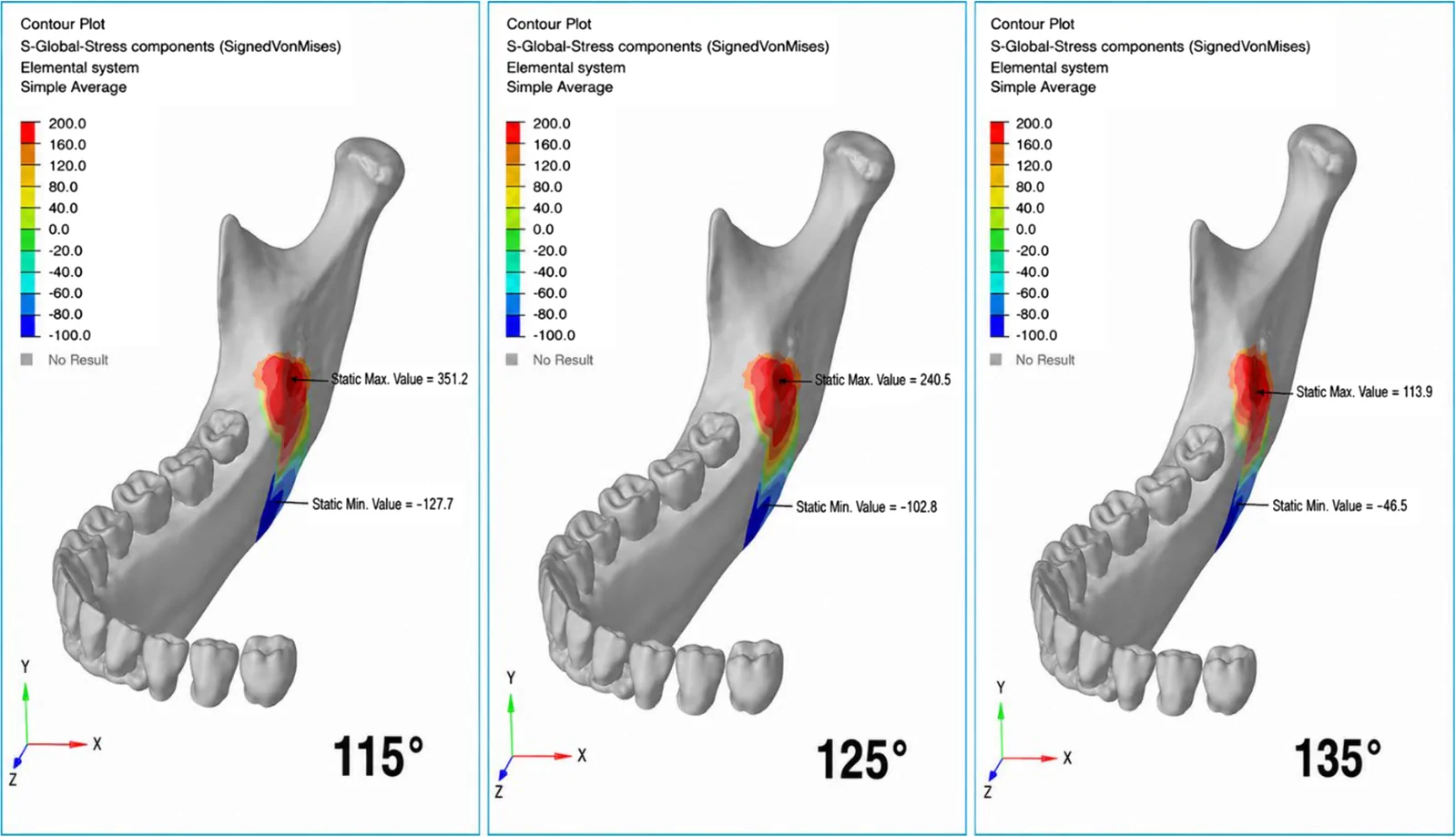
Signed von Mises stress distribution after lateral loading (2000 N) applied to the angulus region in mandibular models with different gonial angles (115°, 125° and 135°)

Under a 2000 N lateral load applied to the angulus region, the highest stress concentration was observed in the model with a 115° gonial angle. As the gonial angle increased, the stress level gradually decreased; in the 125° model, the stresses decreased to a moderate level, and in the 135° model, the peak stress values decreased significantly. While the Pmax and Pmin distributions showed a decrease in tensile and compressive stresses in the order of 115° → 125° → 135°, the Signed Von Mises maps revealed a more intense stress concentration at the angulus line in the 115° model, as summarized in Table 4 and illustrated in Figs. 9, 10, 11 and 12.

**Table 4:** Pmax, pmin, and signed von Mises stresses obtained after lateral loading (2000 N) in the angulus region

Model (Gonial angle)	Pmax (MPa)	Pmin (MPa)	Signed Von Mises (MPa) (+) MPa (−) MPa	
115° model	367.0	−101.0	351.2	-127.7
125° model	253.4	−89.5	240.5	-102.8
135° model	117.9	−34.9	113.9	-46.5

The signed von Mises values represent the von Mises equivalent stress, indicated by a (+/−) sign depending on the direction of the dominant principal stress. Therefore, both positive (maximum) and negative (minimum) values were reported separately in the analysis results

### Evaluation of the bending behavior and possible biomechanical basis of fracture initiation in the mandibular angulus region

The angulus region typically exhibits bending under load. In this biomechanical state, crack initiation primarily occurs on the tensile side (usually the lateral-superior cortex), and it can progress across the neutral axis towards the compressive side (inferior and/or medial cortex). The mechanical basis for these results lies in the fact that as the gonial angle decreases, the angulus region behaves like a sharp corner exhibiting geometric discontinuity. In models with a narrow angle, where the bending moment remained similar, the tensile stresses increased at the cut edge, resulting in a significant stress concentration. In contrast, with a wide gonial angle, the angulus region behaves like a more rounded transition zone, reducing stress concentration and balancing the stress distribution. Therefore, in mandibles with a narrow gonial angle, the tensile stress concentration increases, facilitating crack initiation; crack propagation is affected by the magnitude of the applied load, pattern of repetitive loading, and the microstructural characteristics of the bone tissue.

## Discussion

A mandibular angulus fracture is defined as a fracture line extending from the retromolar region distal to the second molar to any point along the curved line formed by the inferior and posterior borders of the ramus. This region is anatomically predisposed to fracture because it includes the junction of the mandibular body and ramus [6]. Our finite element analysis findings showed that the angulus region exhibited typical bending behavior under mechanical loading, suggesting that the fracture line arises mostly from tensile stress accumulation starting in the lateral-superior cortex and progressing across the neutral axis towards the inferior and medial cortex. This supports the tendency of mandibular angulus fractures to originate from areas of tensile stress and the critical role of the buccal cortex. Furthermore, the angulus region acts as a biomechanical transition zone between the dentate and edentulous segments of the mandible.

As Moore emphasized, sudden changes in bone orientation can lead to structural weakness in the angulus region and, increase susceptibility to fractures. In addition, factors such as soft tissue mass, direction/intensity of the traumatic force, and nature of the impact can significantly influence fracture occurrence [23].

The gonial angle is not merely a measurement but a fundamental indicator of the overall morphology of the mandible and can have significant effects on growth patterns and changes in the facial profile [24]. In the literature, the mean gonial angles in panoramic and cephalometric radiographs have been reported as 127.07 ± 6.10 and 127.5 ± 6.67, respectively; the lack of a significant difference between the two methods supports the clinical accuracy of panoramic measurements [24, 25]. Katti et al. reported that the gonial angle in young adults is mostly in the range of 121°–125° [26]. Panneerselvam et al. defined the normative mean as 121.8 ± 6.2°; classified gonial angles above 128° as high and below 115.6° as low [7].

In our study, a gonial angle of 125° was accepted as the reference value, and three models were created at 115°, 125°, and 135° according to clinical tolerance ranges to comparatively evaluate the effect of gonial angle variations on stress distribution in the angulus region. However, the models were created only with angular modifications of the same mandibular geometry, and other morphological differences, such as ramus height, muscle attachment points, and bone density, which may accompany gonial angle changes in real individuals, were not included in the model. Several simplifying assumptions were adopted in the present finite element models. Bone tissues were modeled as homogeneous, isotropic, and linearly elastic materials, whereas real mandibular bone exhibits anisotropic and heterogeneous mechanical behavior. Consequently, the calculated stress values should be interpreted as relative biomechanical indicators rather than exact representations of in vivo tissue behavior.

In addition, the present simulations were performed using a single static load of 2000 N applied under standardized conditions. Real maxillofacial trauma is considerably more complex and may involve dynamic loading, variable force magnitudes, different impact directions, and varying rates of force application. Consequently, the present findings should be interpreted as representing relative biomechanical tendencies under controlled loading conditions rather than the full spectrum of traumatic events encountered in clinical practice.

Studies reporting a positive correlation between the gonial angle and morphology of the angulus region suggest that the geometric features of this region may increase susceptibility to trauma [27]. Hedeshiu et al. applied traumatic forces of 6667 N to the mandible from different regions and showed a particularly high stress concentration in the condyle [19]. Our study, by adapting a similar numerical approach to the angulus region, revealed how only changes in the gonial angle affect stress distribution on the same mandibular geometry. Thus, the findings of clinical radiomorphometric studies showing “high gonial angles” and the findings of finite element analysis showing “intense stress accumulation at narrow angles” can be interpreted together through different mechanisms. In addition, the validity of the present finite element model is supported by its consistency with previously published studies. Similar to earlier FEA investigations, stress concentration was predominantly observed in the mandibular angulus region, particularly under lateral loading. These findings are in consistent with prior reports demonstrating that the angulus region is one of the most mechanically vulnerable areas of the mandible under traumatic forces. Therefore, although the model is based on simplified assumptions, its outputs show qualitative agreement with the existing biomechanical literature, supporting the reliability of the simulation.

The frequent use of 2000 N as a standardized loading magnitude in finite element analysis studies was the primary reason for adopting this value in the present study. In this protocol, a force of 2000 N was applied perpendicular to a circular contact area with a diameter of 1 cm to simulate traumatic loading conditions under controlled parameters. This magnitude represents high-energy traumatic loading rather than physiological biting forces, which are generally lower and more distributed across the dentition [20–22]. Under these standardized loading conditions, a clear relationship between gonial angle variation and stress distribution in the mandibular angulus region was noted. Specifically, in the frontal loading scenario, the highest stress values were recorded in the 115° model (Pmax: 105.3 MPa; signed von Mises: 101.1 MPa), whereas these values decreased in the 125° model (Pmax: 90.6 MPa; signed von Mises: 88.1 MPa) and reached their lowest in the 135° model (Pmax: 41.7 MPa; signed von Mises: 41.4 MPa). A similar trend was observed for the compressive stress values (Pmin), which decreased from − 51.7 MPa in the 115° model to − 19.2 MPa in the 135° model.

This trend was more pronounced in the lateral loading scenario. The 115° model exhibited the highest stress concentrations (Pmax: 367.0 MPa; signed von Mises: 351.2 MPa), followed by the 125° (Pmax: 253.4 MPa; signed von Mises: 240.5 MPa) and 135° models (Pmax: 117.9 MPa; signed von Mises: 113.9 MPa). The compressive stresses (Pmin) also followed a similar decreasing pattern, from − 101.0 MPa in the 115° model to − 34.9 MPa in the 135° model.

These findings indicate that as the gonial angle decreases, stress concentration in the mandibular angulus region increases significantly under both loading conditions. From a biomechanical perspective, this can be explained by the geometric characteristics of the angle region. A narrower gonial angle creates a sharper transition zone between the corpus and ramus, leading to an increased stress concentration owing to geometric discontinuity. In contrast, a wider gonial angle results in a smoother curvature, allowing stress to be distributed over a larger area thereby reducing peak stress values.

Therefore, the observed increase in tensile stress (Pmax) in the lateral superior cortex of the angulus region in the 115° model suggests a higher susceptibility to crack initiation under high-energy traumatic conditions. These results support the hypothesis that gonial angle variation plays a critical role in determining the biomechanical response of the mandible, particularly in terms of stress localization and fracture initiation.

Shroff et al. found no significant association between gonial angle and angle fractures in their clinical data; however, they reported that the presence of an impacted third molar was a strong risk factor [28]. In our study, when the effect of impacted third molars was eliminated, stress accumulation increased in the narrow gonial angle. This difference suggests that the gonial angle may influence fracture pathogenesis not through a single mechanism, but through different biomechanical pathways. While clinical studies have shown that high gonial angles, with reduced ramus height and cortical support, can facilitate fracture development in low-energy traumas [6, 7, 25], our numerical model showed that at a low gonial angle, geometric “sharpening” increased stress concentration in the lateral-superior cortex, facilitating fracture initiation in high-energy traumas. The apparent discrepancy between our finite element findings and previous clinical studies may be explained by an energy-dependent dual-mechanism hypothesis. These findings may help explain the increased susceptibility of individuals with a high gonial angle to mandibular angle fractures. These findings may provide a possible biomechanical explanation for the differing fracture patterns reported in the literature. One potential interpretation is that low gonial angle mandibles may be more susceptible to localized stress concentration under high-energy loading, whereas high gonial angle mandibles may exhibit reduced structural resistance because of reduced cortical bone thickness, decreased masticatory muscle efficiency, and diminished load-bearing capacity. This perspective may help reconcile previously conflicting findings in the literature and provide a more comprehensive framework for understanding the mechanisms of mandibular angle fractures. Nevertheless, this interpretation remains hypothetical and requires validation through clinical and experimental studies. In contrast, the present finite element analysis demonstrated that narrower gonial angles resulted in increased stress concentration in the angulus region, particularly in the lateral-superior cortex. This finding can be explained by geometric factors, as a reduced gonial angle creates a sharper transition zone that acts as a stress concentrator during loading. Such conditions are more likely to be relevant in high-energy trauma scenarios, where mechanical stress exceeds the structural tolerance of bone.

Therefore, rather than being contradictory, these findings suggest that the gonial angle influences fracture susceptibility through two distinct but complementary biomechanical pathways: (i) structural weakness associated with high gonial angles under low-energy conditions, and (ii) stress concentration associated with low gonial angles under high-energy loading. This dual-mechanism perspective provides a more comprehensive understanding of the pathogenesis of mandibular angle fractures.

Panneerselvam et al. stated that displaced fractures are more likely due to strong muscle traction in the low gonial angle group; however, they also observed a more frequent pattern of displaced fractures in the high gonial angle group [7]. Ben Said et al. demonstrated that angular morphology affects stability by showing that significant displacement occurs at lower forces in models with a high mandibular plane angle [29]. Sirin et al. reported that displacement increased in models with wide gonial angles and that bone plate systems failed at lower loads [30]. Our finite element findings showed increased stress accumulation at a narrow gonial angle. This supports the idea that the gonial angle is not a unidirectional indicator of susceptibility to fracture initiation; rather, it can have a multidimensional effect through different mechanistic mechanisms such as “loss of stability at a wide angle” and “stress concentration at a narrow angle.”

Dhara et al. reported that the mean gonial angle in patients with angulus fracture was 128.5 ± 5.4° and that a higher gonial angle was associated with an increased risk [27]. Panneerselvam et al. reported an average gonial angle of 126.8 ± 7.9° in the angulus fracture group and emphasized the association of a higher gonial angle with an increased risk [7]. Al-Dawoody et al. also reported a gonial angle of 127.60º ± 4.93º in the group with angulus fractures, demonstrating that a high gonial angle increases the risk of angulus fractures [31]. When these clinical studies are evaluated together, it can be said that a wide gonial angle may increase the risk of angulus fractures, especially through decreased cortical support and reduced load-bearing capacity. Our study, on the other hand, reveals stress concentration due to geometric discontinuity in a narrow gonial angle, thus opening a different biomechanical window into the pathogenesis. Therefore, the clinical data of “wide angle – high risk” and the numerical findings of “narrow angle – high stress” can be interpreted not as mutually exclusive mechanisms, but as complementary ones.

### Limitations

The findings of this study should be interpreted with consideration to its methodological limitations. First, models based on finite element analysis represent the mechanical behavior of living tissues under numerical assumptions, and the mandibular bone in this study was modeled with isotropic and linearly elastic properties, in accordance with common approaches. The analyses were performed under linear static assumptions, and the dynamic and high-energy characteristics of real maxillofacial trauma were not fully represented. Furthermore, a single loading value (2000 N) was used in the study; the dynamic forces of the masticatory muscles were not included in the model, and interindividual anatomical variations were not reflected in the model by choosing a standard mandibular geometry. Therefore, the results obtained should be interpreted as a biomechanical assessment showing the relative stress distributions between different gonial angle configurations, rather than absolute clinical values.

## Conclusion

Within the limitations of this finite element analysis study, it can be concluded that gonial angle variation significantly influences stress distribution patterns in the mandibular angulus region under trauma loading conditions. Lower gonial angles were associated with increased stress concentrations, particularly in the lateral-superior cortex, suggesting a higher susceptibility to fracture initiation in high-energy trauma scenarios.

These findings indicate that gonial angle morphology may play a critical role in biomechanical behavior of the mandible and could contribute to fracture risk assessment when combined with clinical parameters. However, further experimental and clinical studies are required to validate these results and establish their direct clinical relevance.

## Data Availability

The datasets generated and/or analyzed during the current study are available from the corresponding author on reasonable request.
